# A parasitoid wasp of *Drosophila* employs preemptive and reactive strategies to deplete its host’s blood cells

**DOI:** 10.1371/journal.ppat.1009615

**Published:** 2021-05-28

**Authors:** Johnny R. Ramroop, Mary Ellen Heavner, Zubaidul H. Razzak, Shubha Govind

**Affiliations:** 1 Biology Department, The City College of the City University of New York, New York, New York, United States of America; 2 PhD Program in Biology, The Graduate Center, New York, New York, United States of America; 3 PhD Program in Biochemistry, The Graduate Center, New York, New York, United States of America; Illinois State University, UNITED STATES

## Abstract

The wasps *Leptopilina heterotoma* parasitize and ingest their *Drosophila* hosts. They produce extracellular vesicles (EVs) in the venom that are packed with proteins, some of which perform immune suppressive functions. EV interactions with blood cells of host larvae are linked to hematopoietic depletion, immune suppression, and parasite success. But how EVs disperse within the host, enter and kill hematopoietic cells is not well understood. Using an antibody marker for *L*. *heterotoma* EVs, we show that these parasite-derived structures are readily distributed within the hosts’ hemolymphatic system. EVs converge around the tightly clustered cells of the posterior signaling center (PSC) of the larval lymph gland, a small hematopoietic organ in *Drosophila*. The PSC serves as a source of developmental signals in naïve animals. In wasp-infected animals, the PSC directs the differentiation of lymph gland progenitors into lamellocytes. These lamellocytes are needed to encapsulate the wasp egg and block parasite development. We found that *L*. *heterotoma* infection disassembles the PSC and PSC cells disperse into the disintegrating lymph gland lobes. Genetically manipulated PSC-less lymph glands remain non-responsive and largely intact in the face of *L*. *heterotoma* infection. We also show that the larval lymph gland progenitors use the endocytic machinery to internalize EVs. Once inside, *L*. *heterotoma* EVs damage the Rab7- and LAMP-positive late endocytic and phagolysosomal compartments. Rab5 maintains hematopoietic and immune quiescence as *Rab5* knockdown results in hematopoietic over-proliferation and ectopic lamellocyte differentiation. Thus, both aspects of anti-parasite immunity, i.e., (a) phagocytosis of the wasp’s immune-suppressive EVs, and (b) progenitor differentiation for wasp egg encapsulation reside in the lymph gland. These results help explain why the lymph gland is specifically and precisely targeted for destruction. The parasite’s simultaneous and multipronged approach to block cellular immunity not only eliminates blood cells, but also tactically blocks the genetic programming needed for supplementary hematopoietic differentiation necessary for host success. In addition to its known functions in hematopoiesis, our results highlight a previously unrecognized phagocytic role of the lymph gland in cellular immunity. EV-mediated virulence strategies described for *L*. *heterotoma* are likely to be shared by other parasitoid wasps; their understanding can improve the design and development of novel therapeutics and biopesticides as well as help protect biodiversity.

## Author summary

Parasitoid wasps serve as biological control agents of agricultural insect pests and are worthy of study. Many parasitic wasps develop inside their hosts to emerge as free-living adults. To overcome the resistance of their hosts, parasitic wasps use varied and ingenious strategies such as mimicry, evasion, bioactive venom, virus-like particles, viruses, and extracellular vesicles (EVs). We describe the effects of a unique class of EVs containing virulence proteins and produced in the venom of wasps that parasitize fruit flies of *Drosophila* species. EVs from *Leptopilina heterotoma* are widely distributed throughout the *Drosophila* hosts’ circulatory system after infection. They enter and kill macrophages by destroying the very same subcellular machinery that facilitates their uptake. An important protein in this process, Rab5, is needed to maintain the identity of the macrophage; when Rab5 function is reduced, macrophages turn into a different cell type called lamellocytes. Activities in the EVs can eliminate lamellocytes as well. EVs also interfere with the hosts’ genetic program that promotes lamellocyte differentiation needed to block parasite development. Thus, wasps combine specific preemptive and reactive strategies to deplete their hosts of the very cells that would otherwise sequester and kill them. These findings have applied value in agricultural pest control and medical therapeutics.

## Introduction

Parasitoid (parasitic) wasps have an obligatory relationship with their insect hosts. Engaged in a biological “arms race,” each partner continuously adapts to the other to emerge alive. For reproductive success, parasitic wasps target their hosts’ behavior, development and immune system. Their attack mechanisms range from biochemical warfare and mimicry, to passive evasion and active immune suppression [1–3]. *Drosophila* and their parasitic wasps are an emerging model for studying how wasps evade or suppress host defenses [4,5]. The generalist *Leptopilina heterotoma* (*Lh*) succeeds on the *Drosophila* species within and beyond the melanogaster group. Its close relative, *L*. *boulardi* (*Lb*), considered a specialist, mainly infects flies of the melanogaster group. Both wasps are highly successful on *D*. *melanogaster*; they consume its developing larval and pupal stages to emerge as free-living adults [6].

Oviposition into second-to-early-third *D*. *melanogaster* larval hosts by *Lb* and *Lh* wasps yields divergent immunological effects. *Lb* infection activates many components of humoral and cellular immunity: Toll-NF-κB, JAK-STAT, and the melanization pathways and their target genes are transcriptionally upregulated; there is a burst of hematopoietic proliferation and differentiation of blood cells (also called hemocytes) in the lymph gland and in circulation. If the immune responses are strong and sustained, macrophages and lamellocytes encapsulate and kill wasp eggs [7–11]. *Lh* infection in contrast suppresses immune gene expression and kills immature and mature larval hemocytes [12,13].

*Lb* and *Lh* females (and also *L*. *victoriae*, a sister species of *Lh*) produce discrete immune-suppressive extracellular vesicle- (EV) like structures in their venom glands (called multi-strategy extracellular vesicles, MSEVs in *Lh* and venosomes in *Lb*) [14,15]. Previously called virus like particles [16–18], these EVs lack clear viral features [15,19]. They are produced in the venom gland, a structure made up of the long gland and a reservoir. The secretory cells of the long gland synthesize and secrete proteins, some of which are initially incorporated into discrete non-spiked vesicle-like structures. In sister species *Lh* and *Lv*, these structures mature in the reservoir and assume a stellate morphology with 4–8 spikes radiating from the center. Mature EVs are roughly 300 nm in diameter, [14,16,20–22]. Packed with more than 150 proteins, EVs are, in part, responsible for divergent physiological outcomes in infected hosts [15,19,23].

Among the most abundant in the *Lh* EV proteins is a 40 kDa surface/spike protein (SSp40) [20]. SSp40 shares structural similarities with the IpaD/SipD family of proteins of the gastroenteric disease-causing Gram negative bacteria, *Shigella* and *Salmonella* [15]. Similar to SSp40’s localization to *Lh* EV spike tips, IpaD localizes to the tips of the T3 secretion injectisome, a bacterial transfer system that injects virulence proteins into mammalian cells. IpaD itself promotes apoptosis of mammalian macrophages [24,25]. These parallels between *Lh* SSp40 and bacterial IpaD/SipD suggest that *Lh* EVs may share some similarities with bacterial secretion systems. Comparative transcriptomic/proteomic approaches revealed that SSp40 and a few other EV proteins are not expressed in the *Lb* venom [15].

Whereas *Lh* EVs lyse lamellocytes within a few hours of wasp attack, *Lb* EVs do not have the same effect [7,16,26]. Our immune-inhibition experiments suggested that *Lh*’s SSp40 mediates EV interactions with lamellocytes [20]. *Lh* infection also uniquely promotes apoptosis of larval macrophages and of lymph gland hemocytes [13]. Macrophages make up more than 95% of all hematopoietic cells while differentiated lamellocytes are rarely found in naïve hosts [8–10,27]. Work in the field strongly suggests that the protein activities concentrated within the *Lh* EVs are responsible for the destruction of these mature and immature blood cells. However, how a macro-endoparasite targets the hematopoietic system and accesses its progenitor population has not been studied. The modes of *Lh* EV entry into these cells and the pathways of destruction are also not well understood.

**Fig 1:**
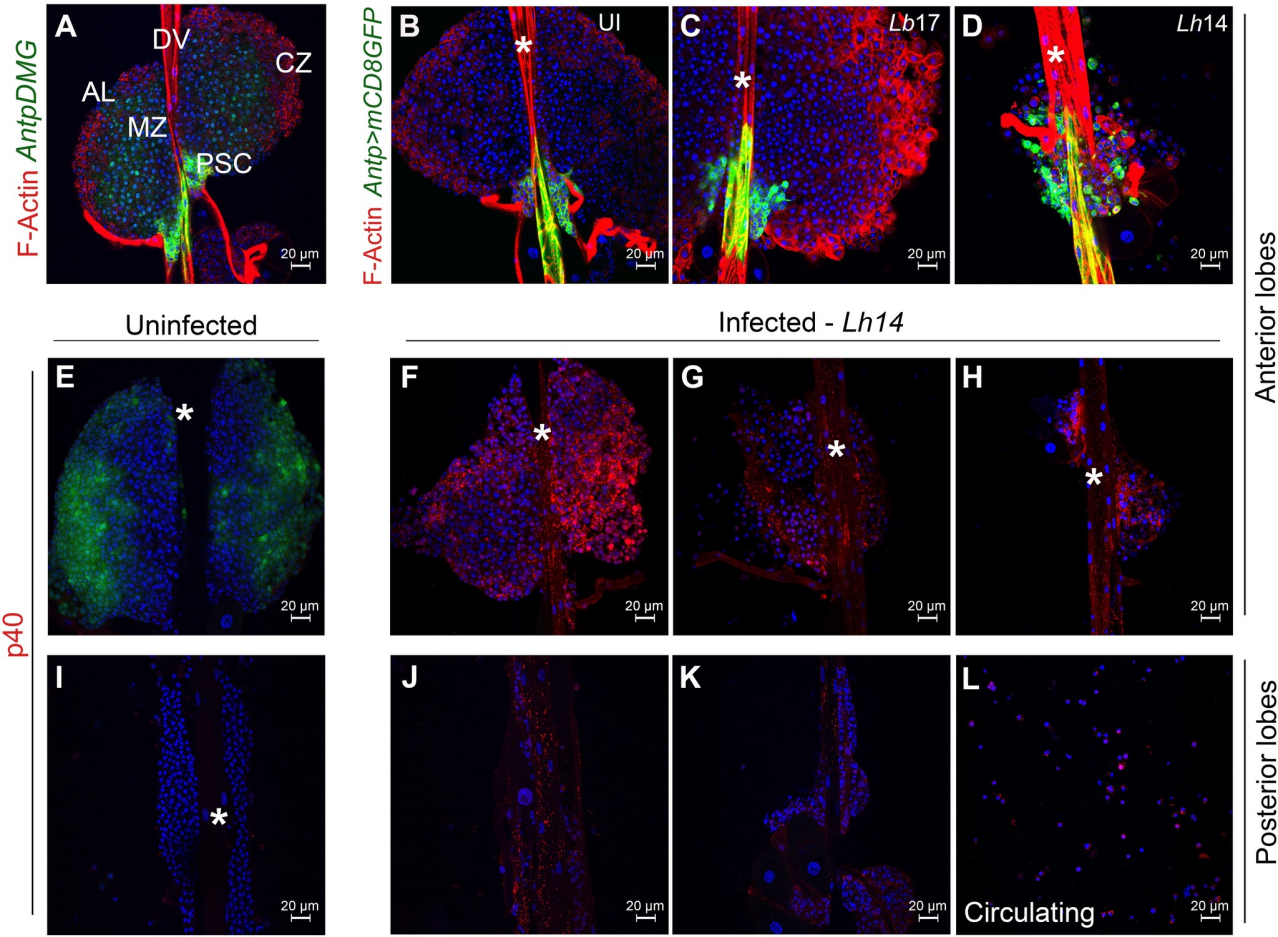
*Lh* EVs associate with the larval lymphatic system. (**A**) Anterior lobes (ALs, without lamellocytes) from a naïve *Antp>mCD8GFP Dome-MESO-GFP* (*AntpDMG*) animal shows medullary zone (MZ), cortical zone (CZ) and posterior signaling center (PSC). The lobes flank the dorsal vessel (DV, asterisk in other panels). (**B-D**) Anterior lobes from uninfected (UI; **B**), *Lb*- and *Lh*-infected (**C, D**) *Antp>mCD8GFP* animals. *Lb17* infection induces lamellocyte differentiation in the cortex (lamellocytes are larger than their progenitors and are rich in F-actin). The GFP-positive PSC appears unaffected. *Lh14* attack leads to loss of lobe cells; the PSC cells are not tightly-clustered and displaced from their original location. (**E-K**) Lymph glands from *Pxn>GFP* animals. (**E**) GFP is expressed in the cortex of uninfected animals, but GFP expression is reduced after wasp attack (**F-H**). *Lh14*-infected ALs from the same infection experiment showing variability in loss of cells; *Lh* EVs (anti-SSp40 staining, referred to as p40 here and in remaining figures) are seen in some cells of these lobes and within the dorsal vessel. (**I-K**) Posterior lobes from uninfected (**I**) and *Lh*-infected animals (**J, K**). (**L**) Circulating hemocytes from infected animals.

The goal of this study was to obtain a macro-level view of *Lh* EV interactions with cells of the larval hemolymphatic system after infection. The term hemolymph refers to the interstitial fluid that distributes hormones, peptides and other macromolecules into organs through the pumping action of an unbranched tubular heart, or the dorsal vessel. Dozens of macrophages circulate in the hemolymph. The heart lumen is surrounded by a column of paired cardiomyocytes and associated pericardial cells. This tubular structure is held in place by alary muscles [28–30]. Hematopoietic cells are organized in paired cell clusters (or lobes) on the dorsal vessel. In third instar larvae, the anterior-most lobes have blood cells at various stages of differentiation; the least differentiated progenitors are confined adjacent to the dorsal vessel, whereas the developing macrophages are sequestered in the cortical regions of the lobes (Fig 1A). In naïve hosts, the progenitor state is maintained by a putative niche (also called the posterior signaling center, PSC). The PSC is a tight unit of about 25–50 cells and is positioned posteriorly to the progenitors [31,32]. Upon *Lb* infection, the PSC reprograms hematopoiesis inducing macrophage and lamellocyte differentiation [30,32–39]. The entire structure is covered by the acellular basement membrane [28,40].

Using *Drosophila* genetics, cell-specific markers and SSp40 staining as a proxy for *Lh* EV localization, we have pieced together a broad view of this host-parasite interaction interface. We show an abundance of *Lh* EVs in (a) the lumen of the larval dorsal vessel, (b) along the collagen/perlecan-based basement membrane around the dorsal vessel and surrounding clusters of lymph gland progenitors, and (c) inside the progenitor and mature macrophages. Moreover, high EV signal correlates with the disassembly of the cohesive PSC unit. PSC ablation limits EV internalization and loss of lymph gland hemocytes, while PSC inactivation via *hedgehog* (*hh*) knockdown (KD) does not have this effect. We also show that lymph gland hemocytes can phagocytose *Lh* EVs using the classical Rab5-mediated retrograde transport (RGT) pathway. Surprisingly, Rab5 also maintains fly macrophage identity, as *Rab5* KD leads to over-proliferation, lamellocyte differentiation and tumorigenesis; *Rab5* function is cell-autonomous. Thus lymph glands are not merely a source of mature blood cells but are themselves immune competent organs and can clear the immune-suppressive *Lh* EVs to defend the host. However, *Lh* EVs proactively dislodge cells of the PSC, blocking differentiation of the protective immune cells. *Lh* EVs target the endomembrane system of macrophages that ultimately results in their apoptosis, thus highlighting central and previously unrecognized roles of the lymph gland in cellular immunity. These observations help explain why *Lh* infections target the larval lymph gland. The direct EV-macrophage interactions and cellular outcomes set the stage for future molecular analyses in both the hosts and parasites.

## Results

### *Lh* EVs are present within the larval lymphatic system

*Lb17* attack triggers lamellocyte differentiation in the larval lymph gland cortex (Fig 1A–1C). At an equivalent time-point, *Lh*-infected lobes are significantly smaller (Fig 1D–1K); [13]. Surprisingly, unlike *Antp>mCD8GFP*-expressing PSCs of naïve and *Lb-*infected lobes that remain tightly clustered (Fig 1A–1C), PSC cells of *Lh*-infected hosts are dislodged and some are distributed in the body of the lobe (Fig 1D).

To understand these responses, we imaged more than 25 hosts in multiple experiments. Throughout these studies, we used a polyclonal antibody to mark SSp40, an *Lh* EV-specific protein [20]. *Lh* infection of *Pxn>GFP* animals reduced *Pxn>GFP* expression (Fig 1E–1H). (*Pxn* is normally active in the cortex and its expression mimics that of many other genes downregulated by *Lh* infection [7]). An abundance of *Lh* EVs was observed in anterior- and posterior-lobe hemocytes, in the dorsal vessel, and in circulating hemocytes (Fig 1F–1H and 1J–1L). This staining signal is absent in glands from naïve animals (Fig 1E and 1I). Thus, *Lb* and *Lh* attack have drastically different outcomes and *Lh* EVs appear to interact directly with most lymph gland hemocytes.

In our analyses across experiments, we found that the degree of tissue loss and EV distribution varies. Lobe morphologies ranged from nearly intact and filled with EVs (Fig 1F and 1J) to damaged lobes, with few to many EVs (Fig 1G, 1H and 1K). This variation is likely due to (a) the duration of infection (i.e., time between oviposition and dissection); (b) the injected EV dose; or (c) the dynamics of EV circulation. Dissections at later time points showed loss of almost all lymph gland hemocytes [13].

**Fig 2:**
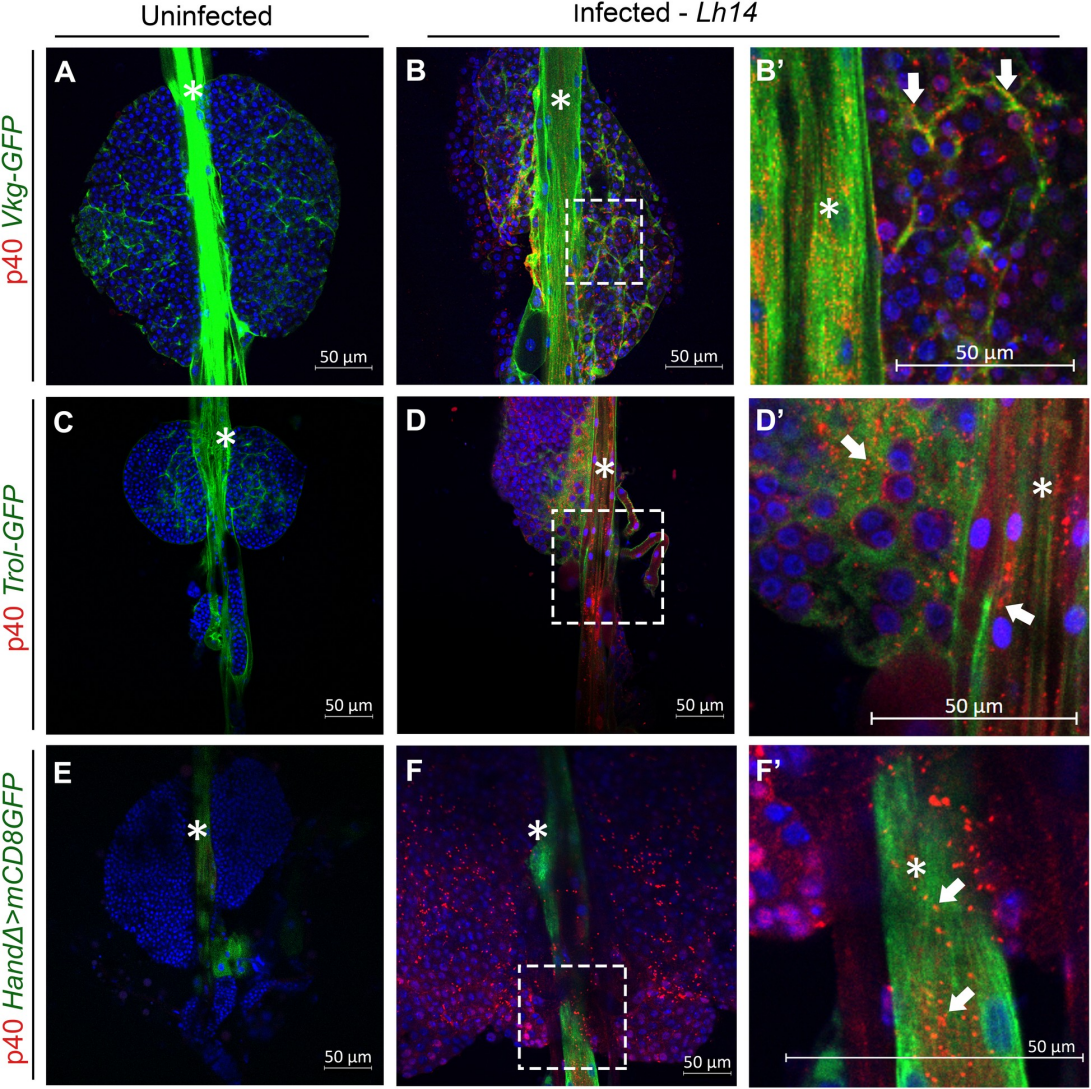
*Lh* EVs associate with basement membrane proteins. (**A-B**) *Vkg-GFP* lymph glands. (**A**) GFP marks Collagen IV in the dorsal vessel and around lobe clusters. (**B-B’**) *Lh*-infected gland shows extensive EV puncta co-localized with GFP in the dorsal vessel (*), and in between lobe clusters. Punctate, cytoplasmic staining in many cells throughout the anterior lobes (**B, B’**, arrows) is also observed. (**C-D**) *Trol-GFP* lymph glands. GFP marks Trol/perlecan distribution in naïve (**C**) and *Lh*-infected (**D**) animals. Co-localization of *Trol-GFP* and EV puncta is observed (**D, D’**, arrows). (**E-F**) *HandΔ>mCD8GFP* marks the cells of the dorsal vessel. (**E**) An intact lymph gland from a naïve animal. (**F, F’**, arrows) EV signal localizes with the GFP signal in the cardiomyocytes.

To then probe how *Lh* EVs enter the lymphatic system, we stained *Lh*-infected glands from fly strains with GFP-tagged Collagen IV (basement membrane, *Viking* [41]) or GFP-tagged proteoglycan core protein, Perlecan/Trol [42]. In both cases, EV puncta were clearly localized with the continuous GFP signals of these extracellular matrix (ECM) proteins along the dorsal vessel as well as in the interstitial spaces around clustered hematopoietic progenitors (yellow puncta in Fig 2A–2D’ arrows). Surprisingly, punctate staining was also seen inside immature progenitors, adjacent to the dorsal vessel (Fig 2B, 2B’ 2D and 2D’). EVs were also observed inside some cardiomyocytes as evidenced by SSp40 colocalization with the mCD8GFP signal in *HandΔ>mCD8GFP* larvae (Fig 2E–2F’, arrows). Thus, *Lh* EVs associate with ECM proteins of the lymphatic system, enter the dorsal vessel lumen and even some cardiomyocytes.

### Effects of *Lh* infection on PSC integrity

Previous studies have demonstrated that upon *Lb* infection, the PSC reprograms hematopoietic development and promotes lamellocyte differentiation. Similar to controls, *Lb* infected PSCs remain tightly clustered [35,37,38,43,44]. We found that regardless of the *Lh* strain, PSC cells dislodged from their normal posterior position into the body of the lobe (Figs 1D and S1A and S1B). At the time points we examined, half of the PSC cells relocated into the lobe, of which an overwhelming majority (> 95%) were present as single cells or as groups of two cells. The other half remained in their original place, although some were not as tightly clustered (Figs 1D and S1; n = 24 PSCs from 12 lymph glands). As expected, in 14 lobes from naïve controls, all PSC cells were tightly packed. Strikingly, in samples from infected animals, where the PSCs were still intact, EVs congregated in regions adjacent to the PSC, but were never found inside the PSC cells (S1A and S1B Fig).

The Slit ligand, originating from adjacent cardiomyocytes, controls PSC integrity via the Robo receptors in the PSC; Robo2 has the strongest effect [45]. Indeed, the effect of *Lh* infection on the PSC resembles *Slit/Robo2* KD, which promotes PSC disassembly [45] (Figs 1 and S1). A cohesive PSC is important in hematopoietic development as fewer macrophages and crystal cells develop in *Slit*/*Robo2* KD lobes compared to controls. But differing from *Lh* infection, *Slit* KD PSCs are larger and there is no apparent loss of progenitors [45]. In spite of different outcomes in the two conditions, we hypothesized that the initial steps might be shared and that *Lh* EVs might inactivate the Slit-Robo signal, which might explain PSC disassembly.

To test this idea, we infected animals in which the Slit-Robo pathway was manipulated to promote constitutive signaling. We found that neither expressing active Slit nor overexpressing Robo2 altered *Lh* EVs’ ability to disassemble the PSC. *Lh* infection of *HandΔ>Slit-N* animals still promoted PSC disassembly (S2A–S2C’ Fig; n = 16 lobes) even though gain-of-function *Slit-N* [46] reverses the effects of *Slit* KD [45]. Similarly, *Lh* infection bypassed the effects of Robo2 overexpression (*Antp>Robo2-HA*) and promoted PSC disassembly S2D–S2F’ Fig). Many EVs are observed around these PSCs (S3 Fig; n = 16). Thus, either EVs inactivate PSC function independently of the Slit-Robo signal, or they possess redundant mechanisms that disable constitutive Slit-Robo signaling.

### PSC-less lymph glands remain intact

**Fig 3:**
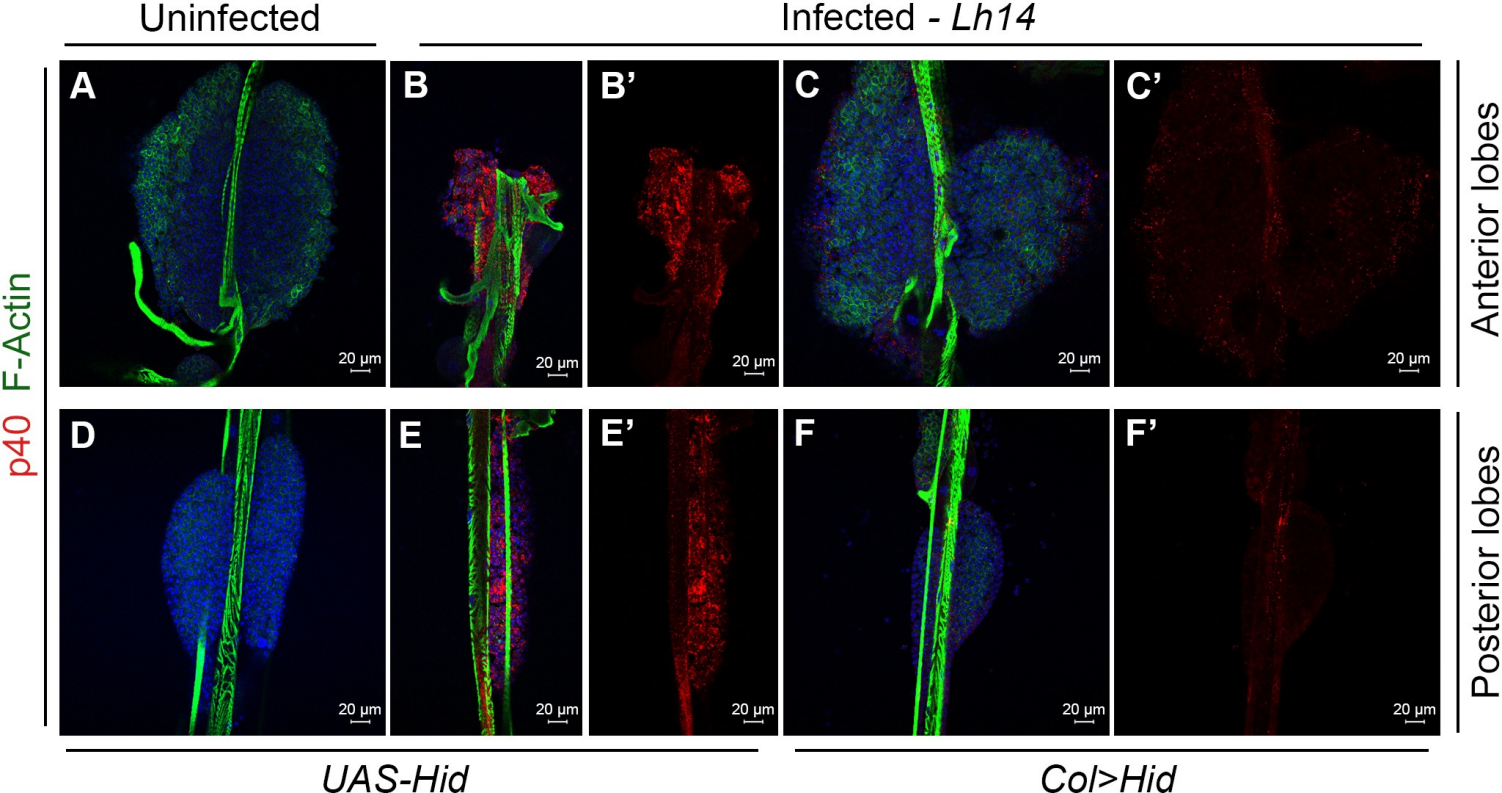
PSC-less lymph glands from *Lh-*infected animals have intact lobes. (**A, D**) Lobes from a naïve *UAS-Hid* host are intact and lack EVs; anterior (**A**), and posterior lobes (**D**). (**B, E**) Lymph gland from a *Lh*-infected *UAS-Hid* host is depleted of hemocytes and has many EVs. Anterior lobes (**B, B’**), and posterior lobes (**E, E’**). (**C, F**) PSC-less lobes from a *Lh*-infected *Col>Hid* host are intact with weak EV signal. Anterior (**C, C’**), and posterior lobes (**F, F’**).

PSC-less lymph glands were unable to induce lamellocyte differentiation after *Lb* infection [36]. We asked if ablating the PSC might similarly inhibit the *Lh* infection responses. PSC-less lobes (*Col>Hid*) lacked Antp staining and *Lh14* infection did not affect lobe integrity (S4A–S4D’ Fig; n > 12 lobes). Moreover, while *Lh*-infected *UAS-Hid* lobes lost progenitors and exhibited high levels of EV uptake (Fig 3A, 3B, 3D and 3E), *Lh*-infected PSC-less lobes remained intact and showed low, non-specific SSp40 staining signal in anterior and posterior lobes (Fig 3C and 3F; n > 12 lobes for each condition). In contrast to the non-responsive *Col>Hid* lobes, *Antp>hh*^*RNAi*^ lobes responded to *Lh* infection and suffered progenitor cell loss (S5A–S5F Fig; n > 12 lobes for each condition). Thus, inactivating the signaling function of the PSC does not appear to affect the wasp’s ability to disassemble the PSC. P1 staining revealed that *Lh* infection does not block macrophage differentiation (S5C Fig). Taken together, these results suggest that the PSC plays a structural role in trafficking of EVs from either the hemolymph or the dorsal vessel into the lobes and that the cell-lethal effects of *Lh* EVs is distinct from the PSC’s niche function.

### The larval lymph gland is phagocytically competent

**Fig 4:**
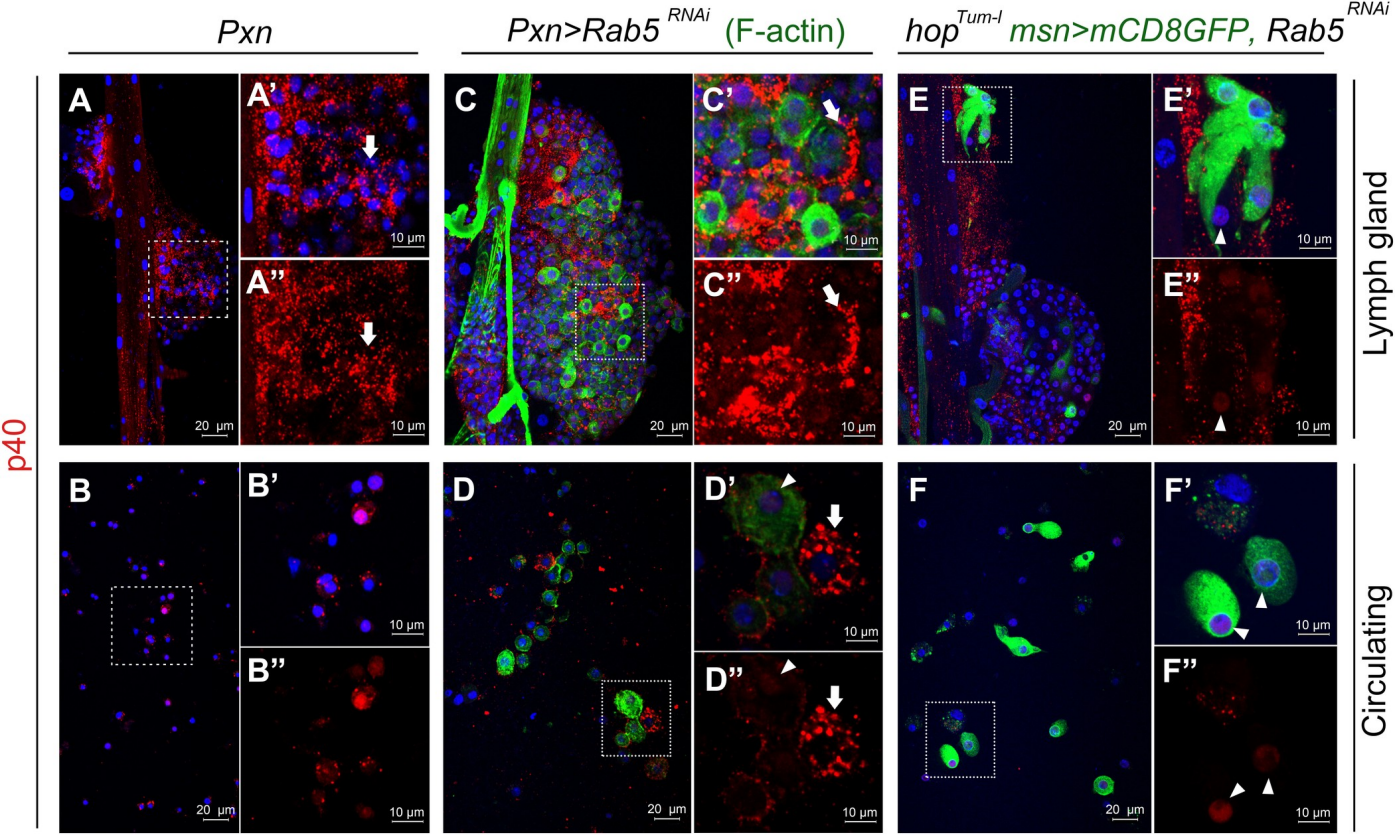
Intracellular *Lh* EV localization. (**A, B**) Anterior lobes (**A-A”)** and circulating hemocytes (**B-B”)** from *Lh*-infected *Pxn>GFP* animals showing EV uptake. Magnifications of areas in (**A**) and (**B**) are shown in panels **A**’, **A**” and **B’**, **B”**, respectively. Arrow points to internalized vesicles. (**C, D**) An anterior lobe (**C-C**”) and circulating hemocytes (**D-D”**) from *Lh*-infected *Pxn>GFP Rab5*^*RNAi*^ animals showing peripheral localization of EVs (arrow). As in Fig 1, *Pxn>GFP* expression is reduced after wasp attack. Samples were counterstained with FITC-Phalloidin to visualize cell morphology. A larger Phalloidin-positive lamellocyte (arrowhead) remains EV negative, while smaller macrophages endocytose EVs. The EV signal is peripheral in some, but not all macrophages (**C, D**). (**E, F**) Anterior l Lobes (**E-E”**) and circulating hemocytes (**F-F”**) from *Lh*-infected GFP-positive lamellocytes of *hop*^*Tum-l*^ *msn>mCD8GFP*, *Rab5*^*RNAi*^ animals. Lamellocytes show a diffuse nuclear SSp40 signal (arrowhead).

We next studied if EV uptake into hematopoietic cells occurs via RGT mechanisms. In *Pxn>GFP*; *+/Bc* heterozygous lymph glands, we observed blackened, dead crystal cells in the cytoplasm of the GFP-positive cortical cells (S6 Fig). This observation suggests that GFP-positive lymph gland cells are phagocytically competent. We therefore investigated if *Lh* EV uptake depends on Rab5, an early endosomal protein. Rab5 mediates trafficking from the plasma membrane to early endosomes [47]. In contrast to *Pxn>GFP* macrophages, where EV staining is bright and punctate throughout the cytoplasm (Fig 4A and 4B), *Pxn>GFP*, *Rab5*^*RNAi*^ cells show peripheral punctate staining, presumably from intact EVs, trapped in early endosomes, both in lymph gland and circulating hemocytes (Fig 4C and 4D; arrows). In lamellocytes, the EV signal is diffuse and nuclear, and *Rab5* KD shows no change in staining intensity or distribution (Fig 4E and 4F; arrowheads), suggesting that Rab5-independent uptake mechanisms are involved. *msn-GFP*- and integrin-β-positive lamellocyte fragments were also observed in *Lh*-infected macrophages suggesting occurrence of efferocytosis (S7 Fig).

### *Lh* EVs negatively impact phagolysosomal organization in macrophages

**Fig 5:**
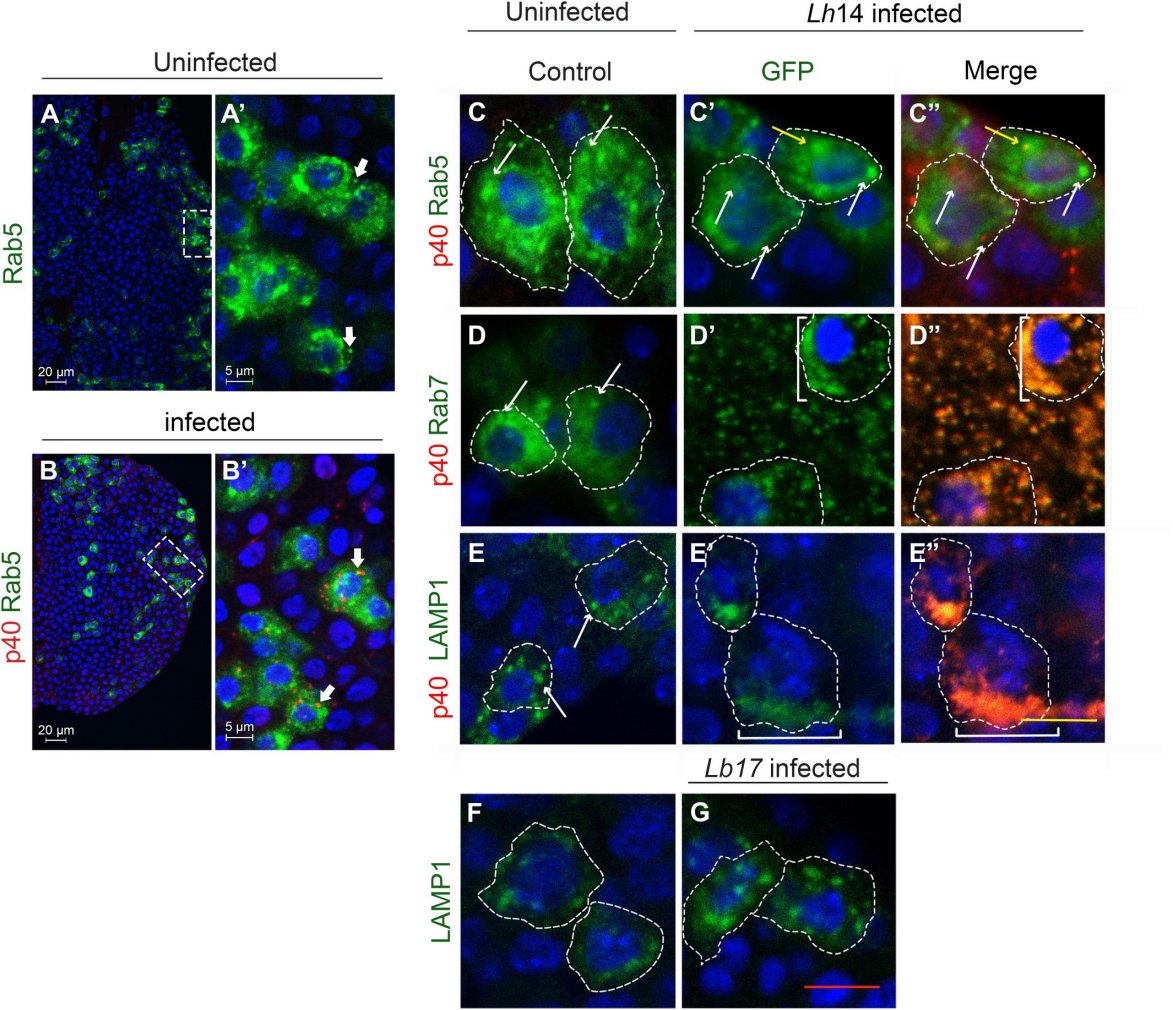
Effects of *Lh* and *Lb* infection on retrograde transport organelles. (**A, B**) *Hemese>GFP-Rab5* lymph glands stained with anti-SSp40; *Lh* EVs enter Rab5 compartments, some EVs colocalize with GFP-Rab5 (arrows). (**C-E**) *Hemese>GFP-Rab5*, *>GFP-Rab7*, and *>GFP-LAMP1* expression in naïve animals or *Lh*-infected animals as shown. Individual cells are outlined. Yellow arrow in C’ and C” shows a normal Rab5 compartment with EV signal. White arrows point to normal compartment morphologies. Square brackets (D’,D”, E’,E”) point to grossly distorted Rab7 and LAMP1 compartments associated with *Lh* EVs. (**F, G**) *Lb* infection does not distort LAMP1 compartment morphologies.

Rab7 mediates late endosome formation and trafficking between late endosomes and lysosomes is marked by Rab7 and LAMP1, respectively [47]. To evaluate if *Lh* EVs impact the RGT machinery, infected glands expressing GFP-tagged Rab5, Rab7, or LAMP1 proteins were examined (Fig 5). Under our experimental conditions, *Lh* EVs rarely colocalized with early endosomes and Rab5 compartment morphology remained comparable to uninfected controls (only 14% of SSp40 puncta are Rab5-positive; n = 221 cells; 6 lobes; Fig 5A–5C). In contrast, *Lh* EVs were consistently found with GFP-Rab7 and GFP-LAMP1 and these compartments were grossly distorted (100% co-localization; n = 115 and 112 cells, Rab7 and LAMP, respectively, 6 lobes each; Fig 5D–5E”). Moreover, the Rab7/EV and LAMP1/EV signals were asymmetrically localized in *Lh*-affected cells. In contrast, *Lb* infection did not distort LAMP-positive compartments and they retained their normal morphology (Fig 5F and 5G). These observations suggest that high numbers of *Lh* EVs transit through early endosomes, but that they are retained in late RGT compartments including lysosomes. Thus, *Lh* EVs have a detrimental effect on RGT compartment integrity and this loss of integrity may promote lysosomal leakage and labilization.

### Rab5 suppresses proliferation and maintains the macrophage fate

**Fig 6:**
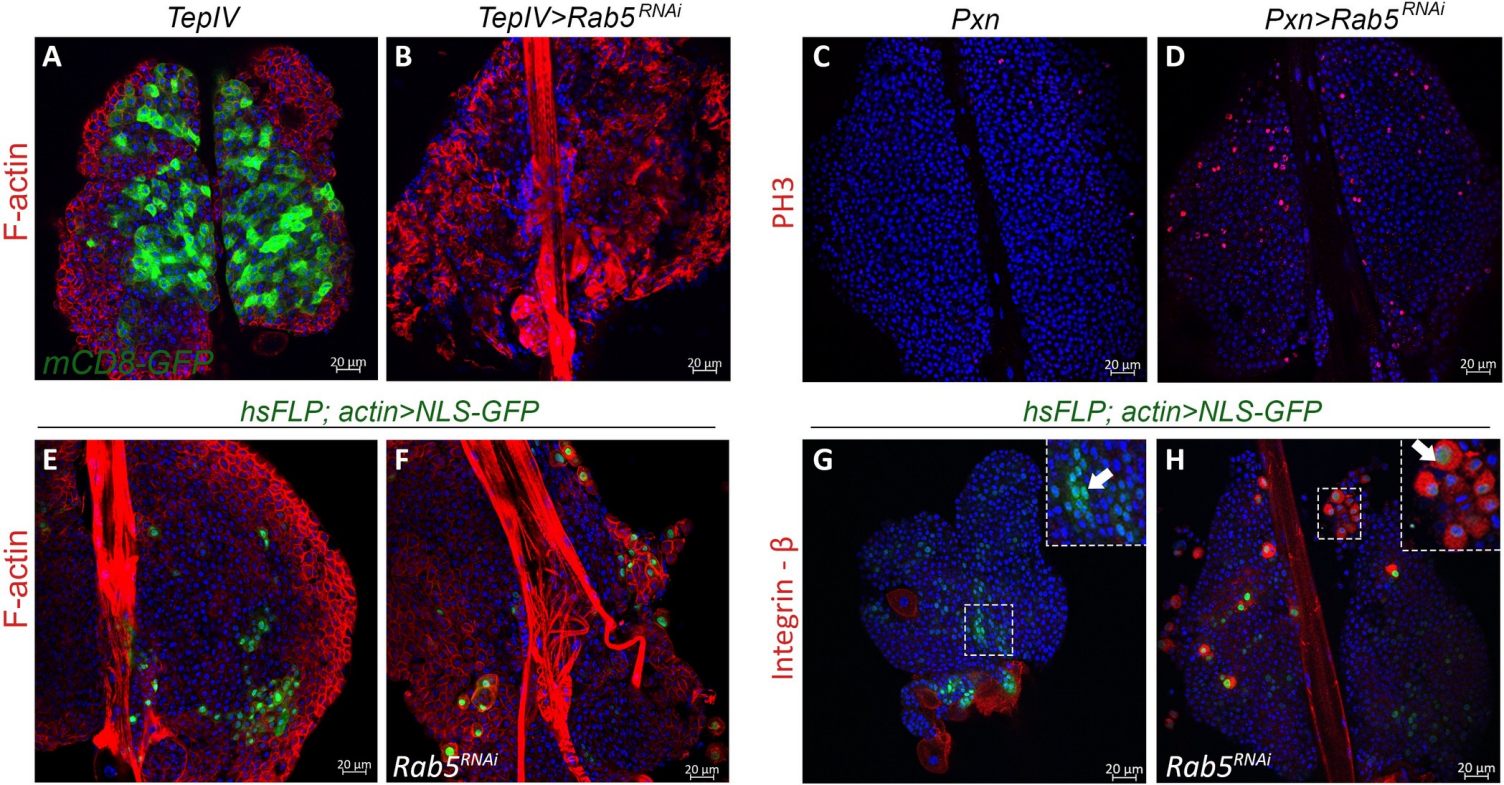
*Rab5*^*RNAi*^ triggers overproliferation and lamellocyte differentiation. (**A, B**) *TepIV>Rab5*^*RNAi*^ in the medullary zone drives lamellocyte differentiation. Lamellocytes are rich in F-actin. (**C, D**) Hemocytes in *Pxn>Rab5*^*RNAi*^ glands exhibit high levels of mitosis marked by phospho-histone3 (PH3) staining. (**E-H**) Cell-autonomous inhibitory role for *Rab5* in lamellocyte differentiation. (**E, G**) *hsFLP; actin>NLS-GFP* control clones (without *Rab5* KD), marked with GFP, do not contain lamellocytes (arrow in panel **G** inset). (**F, H**) *hsFLP; actin>NLS-GFP*, *Rab5*^*RNAi*^ clones have lamellocytes with high levels of F-actin (**F**) and integrin-β (**H**). In panel **H**, the GFP and integrin-β signals overlap in some cells confirming lamellocyte identity (arrow in inset).

We were surprised to find that *Pxn>GFP Rab5*^*RNAi*^ animals developed melanized tumors (S8A and S8B Fig); the hematopoietic population is significantly expanded and lamellocyte differentiation is robust, affecting viability (S8C–S8F Fig). (Variability in viability and tumor development in *Rab5* KD animals is likely due to differences in the strengths and expression patterns of the GAL4 drivers.) A similar result was observed with the expression of the dominant negative Rab5S43N protein which cannot bind GTP [48]). *Rab5* KD even in the lymph gland medullary zone (*TepIV>Rab5*^*RNAi*^) resulted in lamellocyte differentiation (Fig 6A and 6B).

Hematopoietic expansion correlated with increased mitotic index (MI) in lobes of tumor-bearing *Pxn>GFP Rab5*^*RNAi*^ animals (Fig 6C and 6D) suggesting that normal Rab5 function checks over-proliferation and ectopic progenitor differentiation. (MI = 2.2 ± 2.15 in control *Pxn>GFP*; 2.9 ± 1.1, and 5.5 ± 1.9 in experimental *Pxn>GFP*, *Rab5*^*RNAi*^ animals without and with tumors, respectively (n = 10 for each condition). These results suggest that *Rab5* acts as a tumor suppressor and maintains hematopoietic immune quiescence.

Control “FLP-out” clones without *Rab5*^*RNAi*^ contained small cells that did not express integrin-β; experimental clones with *Rab5*^*RNAi*^ had larger, F-actin-rich cells with a typical lamellocyte morphology, that were also integrin-β-positive (Fig 6E–6H). These results suggest that Rab5’s requirement in maintaining the progenitor or macrophage fate is cell-autonomous.

## Discussion

### System-wide distribution but specific effects of *Lh* EVs

**Fig 7:**
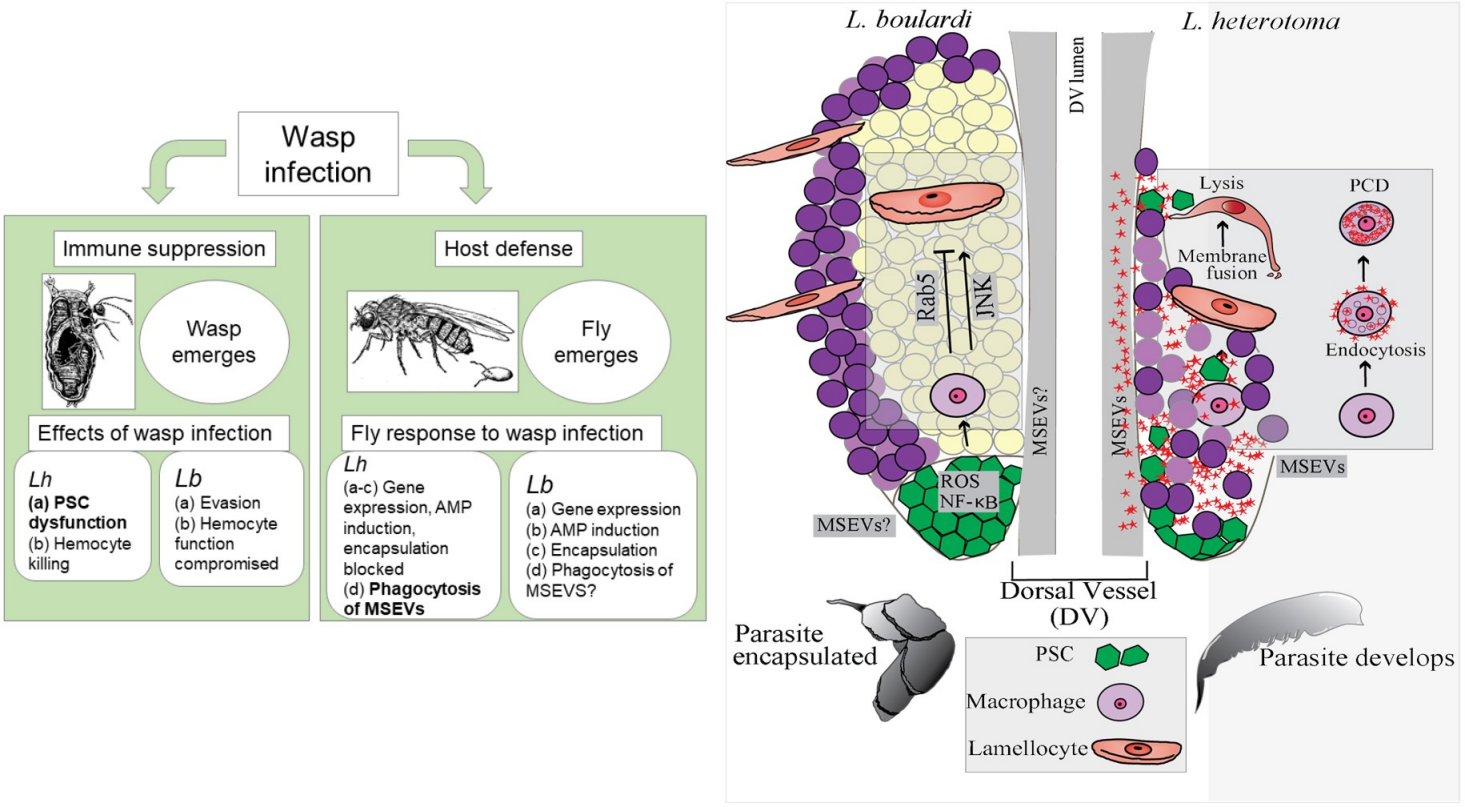
*Lh* EV interactions and effects on host blood cells: summary of events. Left: Immune suppression by *Lh* and *Lb* wasps and host defense in response to infection. Text in bold indicates findings from this study. Right: *Lb* attack triggers NF-κB-dependent signaling events in the PSC and promotes lamellocyte differentiation of lymph gland progenitors (left lobe). This process is important in host defense against parasitic wasps [35,44] and is kept in check by Rab5. After *Lh* infection (right lobe), *Lh* MSEVs concentrate around and disassemble the PSC. They are phagocytosed by macrophages by a Rab5-dependent endocytic mechanism. In macrophages, EVs damage the phagolysosomal compartments. They are internalized by lamellocytes independently of Rab5 function. EVs lyse the few lamellocytes that differentiate post infection.

Parasitism by *L*. *heterotoma* has been of interest because of its ability to parasitize many *Drosophila* hosts and the existence of venom factors that kill host hemocytes. *Lb* lacks these activities (Fig 7). The discovery of an anti-lamellocyte activity intrinsic to *Lh* EVs provided initial insights into the critical roles of these EVs in parasitism [20,49]. However, details underlying their apoptotic effects on macrophages have been lacking. This work provides the first view into how *Lh* EVs rely on the host’s circulation to gain system-wide distribution to not only precisely kill the available effector cells but also to pre-emptively interfere with the host’s ability to produce additional effector cells. We show that lymph glands serve an important, previously unappreciated role in immunity. A majority of lymph gland cells can phagocytose *Lh* EVs to protect the host from their detrimental effects. EV activities in turn promote their apoptotic death by disrupting their endomembrane system.

This work also lays bare new questions. *Lh* EVs’ association with the ECM proteins around the lymphatic system cells suggests ways in which EVs might recognize and home into the lymph gland hemocytes and cardiomyocytes although the role of the ECM, the details of their entry and physiological effects on cardiomyocytes are currently unclear. As has been suggested for Slit-carrying vesicles [45], cardiac cells might provide a route for *Lh* EVs to converge into the vicinity of the PSCs. Once inside lymph gland hemoctytes, they simultaneously target the protective functions of macrophages and lamellocytes and their activities culminate to strongly block encapsulation (Fig 7). These strategies are not uncommon and are likely to be shared by closely related *Leptopilina* wasps or even unrelated virulent wasps that attack drosophilid and non-drosophilid hosts and are known to destroy their hosts’ hematopoietic cells [50–54].

### *Lh* EVs proactively block encapsulation

Studies with *Lb* showed that the lymph gland itself responds to wasp infection and lamellocytes differentiate from hematopoietic progenitors [8,9,55,56]. In addition to PSC’s niche function in naïve animals [31,32,57–59], the PSC also appears to play an anti-parasite role as *Lb* infection promotes lamellocyte differentiation [30,35–38,43,60]. Given this latter role, it is reasonable to interpret *Lh’*s effects on PSC integrity as part of a corresponding adaptive strategy that *Lh* has acquired during its evolutionary history.

The high *Lh* EV levels around the PSC and its disassembly provide novel physiological insights into PSC functions and raise intriguing mechanistic questions. A normally clustered and cohesive PSC organization is needed for proper hematopoietic differentiation in naïve animals [45]. Although *Lh* infection-induced PSC disassembly and hemocyte loss are observed together in fixed samples, our data from infected PSC-less animals suggest that PSC disruption might precede hemocyte death. It is possible that the PSC may somehow “recognize” foreign entities and might serve to protect the progenitor microenvironment by acting as a chemical or mechanical barrier between the vascular and hematopoietic cells. In this scenario, *Lh* EVs may be targeting PSC cohesiveness to inactivate this barrier function.

This interpretation is consistent with the recently discovered permeability barrier in the PSC that is breached by systemic bacterial infection. Permeability barrier in the PSC is maintained by septate junctions and their disruption is linked to increased Toll signaling, cellular immune activation and improved host survival [39]. The barrier function between the vascular and hematopoietic cells proposed here would serve to limit the ingress of structures such as microbes and EVs. The mechanisms underlying the effects of ablated PSCs are unclear but it is notable that PSC-less lobes do not respond to either *Lb* or *Lh* infections (our results and [36]). Examining whether *Lb* EVs/venosomes similarly interact with the lymph gland ECM, congregate around the PSC, and are phagocytosed by hemocytes will shed light on these processes.

Extracellular vesicles secreted from mammalian neutrophils and endothelial cells can direct cell motility and chemotaxis [61,62]. Thus, it is possible that *Lh* EV activities [15] are similarly responsible for PSC disassembly. These activities may perturb host pathways required for normal PSC cohesion and function, although manipulating the Slit-Robo pathway components, or *hh* signaling, hypothesized to disrupt the infection process, was insufficient to block *Lh*’s ability to disperse the PSC and attack hemocytes. *Lh* EVs may possess redundant or independent mechanisms to control PSC integrity and this question remains open for further research.

### A central role for phagocytosis in the anti-parasite response

The ability of macrophages to ingest and kill microbes is a fundamental facet of innate immunity. Microbes have evolved to evade or escape the destructive conditions in their host cells’ phagolysosomes. While most intracellular pathogens avoid fusion with lysosomes, others modify endocytic trafficking differently to survive in their host cells [63]. We have shown that like microbes, *Lh* EVs are endocytosed and can damage the late endocytic compartments. This suggests that their biochemical activities may distort and damage intracellular membranes although how this occurs mechanistically is unclear. A novel family of *Lh* EV-associated GTPases [15] are possible candidates for such activities as expression of select GTPases in yeast alter vacuolar morphologies [64]. Microbial infection of macrophages can activate apoptosis responses [65–67] and it may be that similar effects of *Lh* EVs in fly macrophages are directly linked to their apoptosis.

Lamellocytes utilize a flotillin/lipid raft dependent mechanism to internalize *Lb* EVs [68], and it is likely that *Lh* EVs use the same or a similar pathway. The significance of the nuclear SSp40 signal in lamellocytes after *Lh* infection is unclear; but because the signal is not punctate, *Lh* EVs are likely internalized via a membrane fusion step in which their vesicular character is lost. Electron microscopy results also show that unlike macrophages with membrane-enclosed endocytic vesicles containing intact *Lh* EVs, lamellocytes do not have such compartments, and once internalized, EVs lose structural integrity [49]. Efferocytosis of lysed lamellocytes appears to be an effector anti-parasite response and it may ultimately also be beneficial to parasite development.

Our genetic studies with Rab5 highlight the central role of the endocytic processes in the anti-parasite response. Loss of endocytic trafficking activates immune signaling ([69–71]). From a physiological standpoint, it makes sense why the lymph gland is a dedicated target of wasp infections. Both key aspects of anti-wasp cellular immunity, i.e., phagocytosis of the wasp’s EVs and lamellocyte differentiation, unequivocally reside in the lymph gland. These ideas can be further examined at the molecular level with the available descriptions of the *Lh* and *Lb* EV proteomes [15,19,23]. Virulence factors provide the armament for parasite success in the host/pathogen “arms race”. Insights from this model host-parasite system can influence our understanding of how parasite-derived factors have shaped the immune physiology of fly hosts.

## Materials and methods

### Stocks and crosses

All *D*. *melanogaster* stocks were raised on standard fly medium containing cornmeal flour, sucrose, yeast, and agar at 25°C.

GAL4 lines: PSC drivers were: *Antp-GAL4; mCD8GFP* ([72], from S. Minakhina) and *y w; Collier-GAL4/CyO y*^*+*^ ([32] from M. Crozatier). The truncated *HandΔ* promoter is active in cardiomyocytes of the dorsal vessel ([73], from M. Crozatier). Hemocyte drivers were: *Pxn-GAL4*, *UAS-GFP* ([74], from U. Banerjee); *Hemese-GAL4* (*He-GAL4*) ([75], from D. Hultmark); *eater (ea)-GAL4 (*[76], from R.A. Schulz); *Collagen-GAL4* (*Cg>GFP*) ([77], from C. Dearolf); *Serpent (Srp)-GAL4* [78] and *TepIV-GAL4* [79] (both from N. Fossett); *Hemolectin-GAL4* (*Hml>GFP*) ([80], from J-M. Reichhart).

UAS lines: The *UAS-Slit-N* ([46]; Slit gain-of-function) and *UAS-Robo2-HA* (for overexpression of Robo2, [81]) lines were obtained from T. Volk and T. Kidd.

Strains from the Bloomington Drosophila Stock Center: *UAS-Rab5*^*RNAi*^ (#30518); *UAS-GFP-Rab5* (#43336) [82]; *UAS-GFP-Rab7* (#42706); *UAS-Rab5*.*S43N* (#42704); *UAS-GFP-LAMP*; *nSyb-GAL4/CyO*:*TM6B* (#42714) [83], and *UAS-hh*^*RNAi*^ (#25794) [84]).

Other lines: A homozygous *Bc* stock devoid of other mutations (from B. Lemaitre [85]) was balanced with *CyO-GFP* for crosses with the homozygous *Pxn-GAL4*, *UAS-GFP* strain. Protein trap lines were: *Collagen IV* (Viking) and *perlecan* (*Trol*) (from A. Spradling and L. Cooley). In the *hhf4f-GFP; Antp-GAL4/TM6 Tb Hu* strain, the PSC is marked (from R.A. Schulz). In the *Dome-MESO-GFP* strain, the lymph gland medulla is GFP positive ([86], from M. Crozatier). We recombined this latter insertion with the *Antp-GAL4* insertion to make a *UAS-mCD8GFP; Antp-GAL4*, *Dome-MESO-GFP* (*AntpDMG*) stock. *hop*^*Tum-l*^, *msn-GAL4; UAS-mCD8GFP* [87] uses the *misshapen* (*msn*) driver to mark lamellocytes [88]. For PSC-less animals, *UAS-Hid* [89] females were crossed with *Collier-GAL4/CyO y+* males. For FLP-out clones [90], *hsp70-flp; Actin>CD2>GAL4* flies were crossed with the *Rab5*^*RNA*i^ flies; progeny was heat shocked at 37°C as described [38]. UAS-GAL4 crosses were maintained at 27°C.

### Wasp infections

*y w* flies were used to rear wasps. Unless specified otherwise, infections were done with either *Lb17* or *Lh14* [7]. *LhNY* [20] was used to validate results with the *Lh14* strain. Ten to twelve trained female wasps were introduced to hosts from a 12-hr egg-lay. Hosts were allowed to recover after an 8–12 hr infection. Dissections were typically done one-to-two days after infection. Uninfected controls followed the same timeline. In general, longer infection regimes led to stronger responses: more lamellocytes differentiated after *Lb* infection and more lobe cells were lost after *Lh* infection. Under our experimental conditions, superparasitism by either wasp was rare and for our analyses, we avoided hosts with more than one parasite.

### Immunohistochemistry

Antibody staining was performed according to [91]. Primary mouse anti-SSp40 (1:1000) [20] and Cy3 AffiniPure donkey anti-mouse secondary (1:200) (Jackson Immuno Research) were used to detect *Lh* EVs. Mouse anti-Antennapedia (1:10; Developmental Studies Hybridoma Bank 8C11, [92] and macrophage-specific mouse anti-P1 (1:20; I. Ando [93]) were similarly detected. Nuclear dye (Hoechst 33258, Invitrogen, 1:500) and Rhodamine or Alexa Fluor 488-tagged Phalloidin (Invitrogen) were used for counterstaining cells. For mitotic index, rabbit anti-phospho-histone3 (1:200 Molecular Probes)-positive hemocytes were scored in randomly selected 1000 μm^2^ areas of imaged lobes.

Samples were mounted in VectaShield (Vector Laboratories). Lamellocytes were visualized by (a) high F-actin staining signal, (b) integrin-β (1:200, Developmental Studies Hybridoma Bank CF.6G11 [94]) expression, or (c) *msnf9-GFP* expression [88]. Representative results from twelve or more dissections from at least three independent experiments are presented, unless specified otherwise.

### Confocal imaging

Mounted samples were imaged with Zeiss laser scanning confocal microscopes LSM 510 or LSM 710. For each experiment, images were scanned on the same microscope with the same software and scan settings. Images were gathered at 0.8 μm -1.5 μm and recorded at 8 bit. Laser amplifier gain and offset values were set with negative controls lacking either primary antibodies or wasp infection. Images were processed with Zeiss LSM image browser or Zen Lite 2012. Figures were assembled in Adobe Photoshop v12.0.4 and CC 2015.5 or Illustrator CC 2015.3.

## Supporting information

S1 Fig *Lh* EVs congregate around and disassemble PSCs. (**A, B**) SSp40 staining of lymph glands from *Lh14-* **(A-A”**) or *LhNY-*infected (**B-B”**) *Antp>mCD8GFP* hosts. Strong punctate EV signals are observed around the GFP-positive PSCs and in hemocytes. Areas in the PSC are enlarged in the insets to show details.
(TIF)

S2 Fig *Lh* infection overrides Slit-Robo signaling. (**A-C**) Antp staining of lymph glands from Hand*Δ>mCD8GFP* (**A, A’**) and Hand*Δ>mCD8GFP*, *Slit-N* (**B-C’**) hosts. The tight clustering of Antp-positive PSC in infected hosts is lost and the PSC is disassembled (**C, C’**). (**D-F**) Lymph glands from *Antp>mCD8GFP* (**D, D’**) and *Antp>mCD8GFP*, *Robo2-HA* hosts (**E-F’**). (**E, E’**) Robo2-HA expression tightens the GFP-positive PSC. (**F-F’**). *Lh* attack overrides this effect. *Lh* EVs are associated with these *Antp>mCD8GFP*, *Robo2-HA lobes* (see **S3 Fig**).
(TIF)

S3 Fig *Lh* EVs in *Antp>Robo2-HA* lobes. Anterior lobes of lymph glands from uninfected *Antp>mCD8GFP* (**A, A’**) and *Lh*-infected *Antp>mCD8GFP*, *Robo2-HA* animals (**B, B’**). EVs are absent in glands of naïve animals (**A, A’**) but clearly observed and widely distributed in glands of infected animals. The PSC is no longer tightly clustered. (The sample in panels **B**, **B’** is the same as shown in **S2 Fig**, panels **F, F’**).
(TIF)

S4 Fig PSC-less lymph glands do not respond to *Lh* infection. (**A, A’**) A normal-sized and intact PSC, expresses Antp in *UAS-Hid* animals. Lobes from naïve animals have normal morphology. (**B, B’**) An Antp-positive PSC is disassembled in *UAS-Hid* animals after *Lh* infection. Lobes are reduced in size. Insets in panels A’ and B’ show Antp-positive PSC cells. (**C, D**) A PSC-less lymph gland from *Col>Hid* naïve and *Lh*-infected hosts. Lobes are Antp-negative. *Col>Hid* lobes remain intact after *Lh-*infection (**D, D’**). The dashed lines in panels (**C**) and (**D**) show the areas where biological samples are present. Arrows point to the general locations where the PSCs should have formed.
(TIF)

S5 Fig *Antp>hh*^*RNAi*^ PSCs respond to *Lh* infection. (**A-D**) Lobes from naïve *Antp-GAL4; hhf4f-GFP* (**A, C**) and *Antp>hh*^*RNAi*^*; hhf4f-GFP* (**B, D**) hosts. *hh* KD increased cortical P1-positive cells (**B**); *Lh* infection leads to hemocyte loss and disassembled PSCs. P1-positive cells are observed post-infection (**D**). (**E, F**) Anterior (**E**) and posterior (**F**) lobes from *Lh-*infected *Antp>hh*^*RNAi*^; *hhf4f-GFP* hosts show EVs in the few remaining hemocytes. EVs are also evident in the dorsal vessel.
(TIF)

S6 Fig Blackened crystal cells are phagocytosed by lymph gland hemocytes. (**A, A’**) A *Bc*^*+*^*/Bc Pxn>GFP* gland showing blackened crystal cells within *Pxn>GFP*-expressing hemocytes. Arrows points to crystal cell nuclei; arrowheads point to *Pxn>GFP*-positive macrophages. Not all macrophages contain a crystal cell.
(TIF)

S7 Fig Efferocytosis of disintegrating lamellocytes. Hemocytes from an *Lh*-infected *hop*^*Tum-l*^ host in which lamellocytes (L) express *mCD8GFP*. Lamellocytes also express high levels of integrin-beta. Double positive lamellocyte fragments in panel **A** are observed in macrophages (M) indicated by arrows. Signals in **A’** and **A”** are merged in panel **A**.
(TIF)

S8 Fig Tumorigenesis and lethality in *Rab5* knockdown animals. (**A, B**) *Pxn>GFP*, *Rab5*^*RNAi*^ larvae with melanized tumors. Tumors are absent in the control animal. (**C, D**) Circulating hemocytes from *Rab5* KD animals show an overabundance of *Pxn>GFP-*positive and GFP-negative (lamellocytes) cells. (**E**) Tumor penetrance (animals with tumors/animals scored) in *Rab5* KD animals varied with different GAL4 drivers. (**F**) Viability to adulthood was differentially affected. More than 100 animals were scored for each cross in panels (**E**) and (**F**).
(TIF)
