# Supplementary figures and images for: A parasitoid wasp of Drosophila employs preemptive and reactive strategies to deplete its host’s blood cells
Source: PLoS Pathog. 2021 May 28;17(5):e1009615. doi: 10.1371/journal.ppat.1009615 (PMC8191917; doi:10.1371/journal.ppat.1009615)

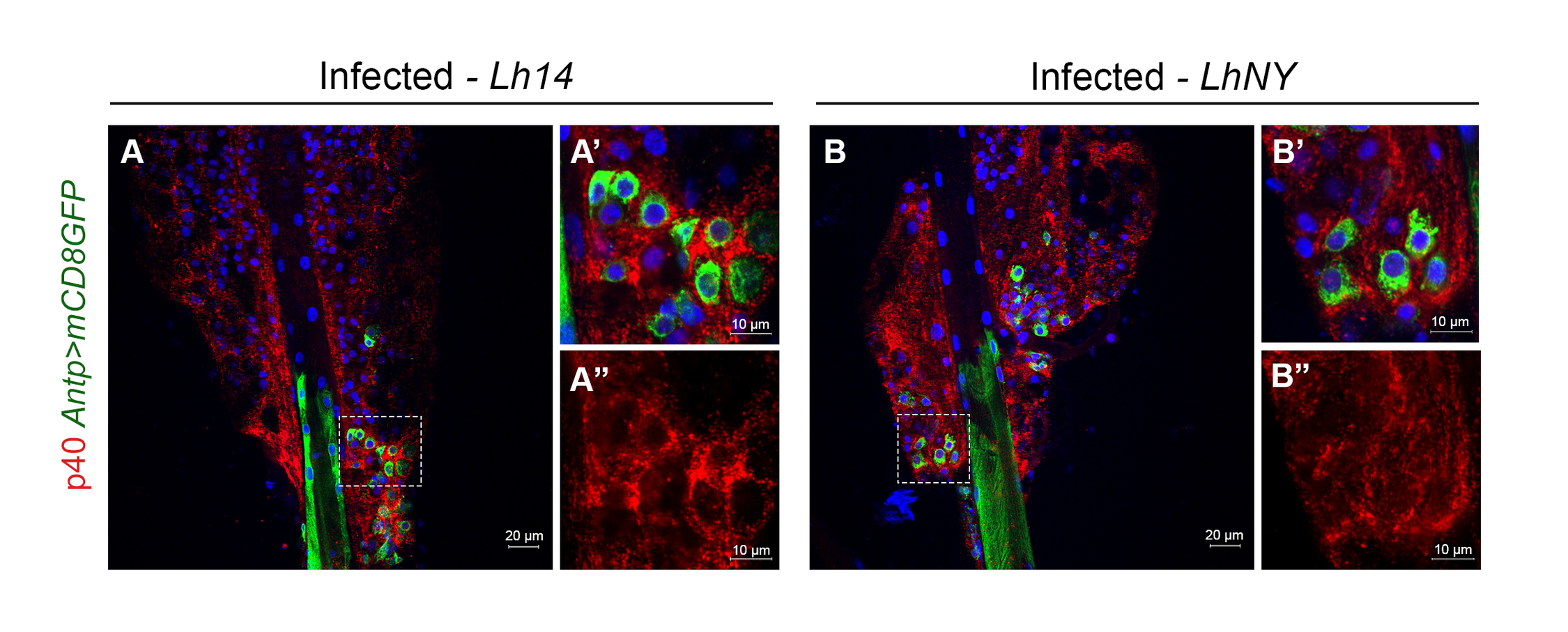

Supplement: S1 Fig — (A, B) SSp40 staining of lymph glands from Lh14- (A-A”) or LhNY-infected (B-B”) Antp>mCD8GFP hosts. Strong punctate EV signals are observed around the GFP-positive PSCs and in hemocytes. Areas in the PSC are enlarged in the insets to show details. (TIF) [file ppat.1009615.s001.tif]

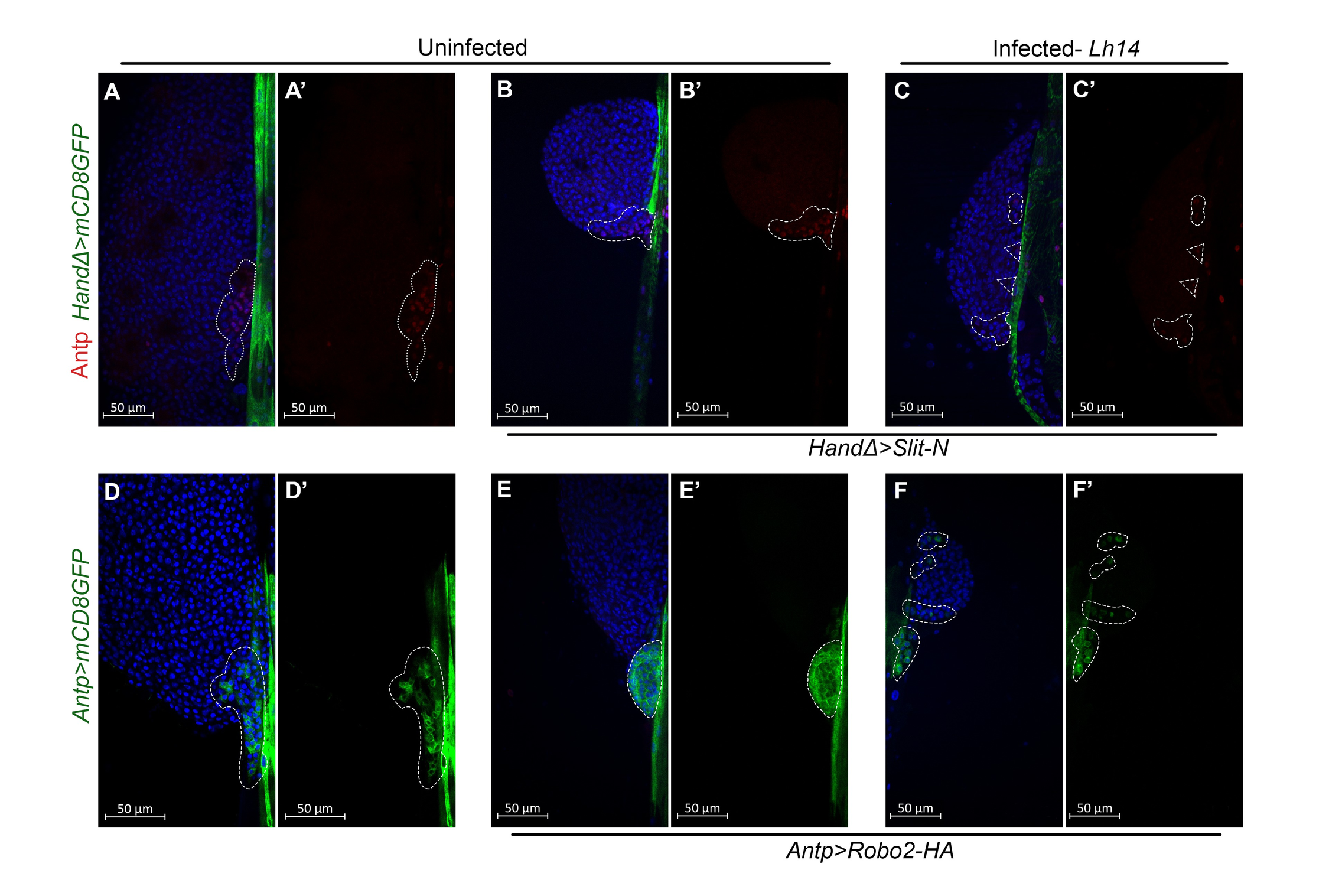

Supplement: S2 Fig — (A-C) Antp staining of lymph glands from HandΔ>mCD8GFP (A, A’) and HandΔ>mCD8GFP, Slit-N (B-C’) hosts. The tight clustering of Antp-positive PSC in infected hosts is lost and the PSC is disassembled (C, C’). (D-F) Lymph glands from Antp>mCD8GFP (D, D’) and Antp>mCD8GFP, Robo2-HA hosts (E-F’). (E, E’) Robo2-HA expression tightens the GFP-positive PSC. (F-F’). Lh attack overrides this effect. Lh EVs are associated with these Antp>mCD8GFP, Robo2-HA lobes (see S3 Fig). (TIF) [file ppat.1009615.s002.tif]

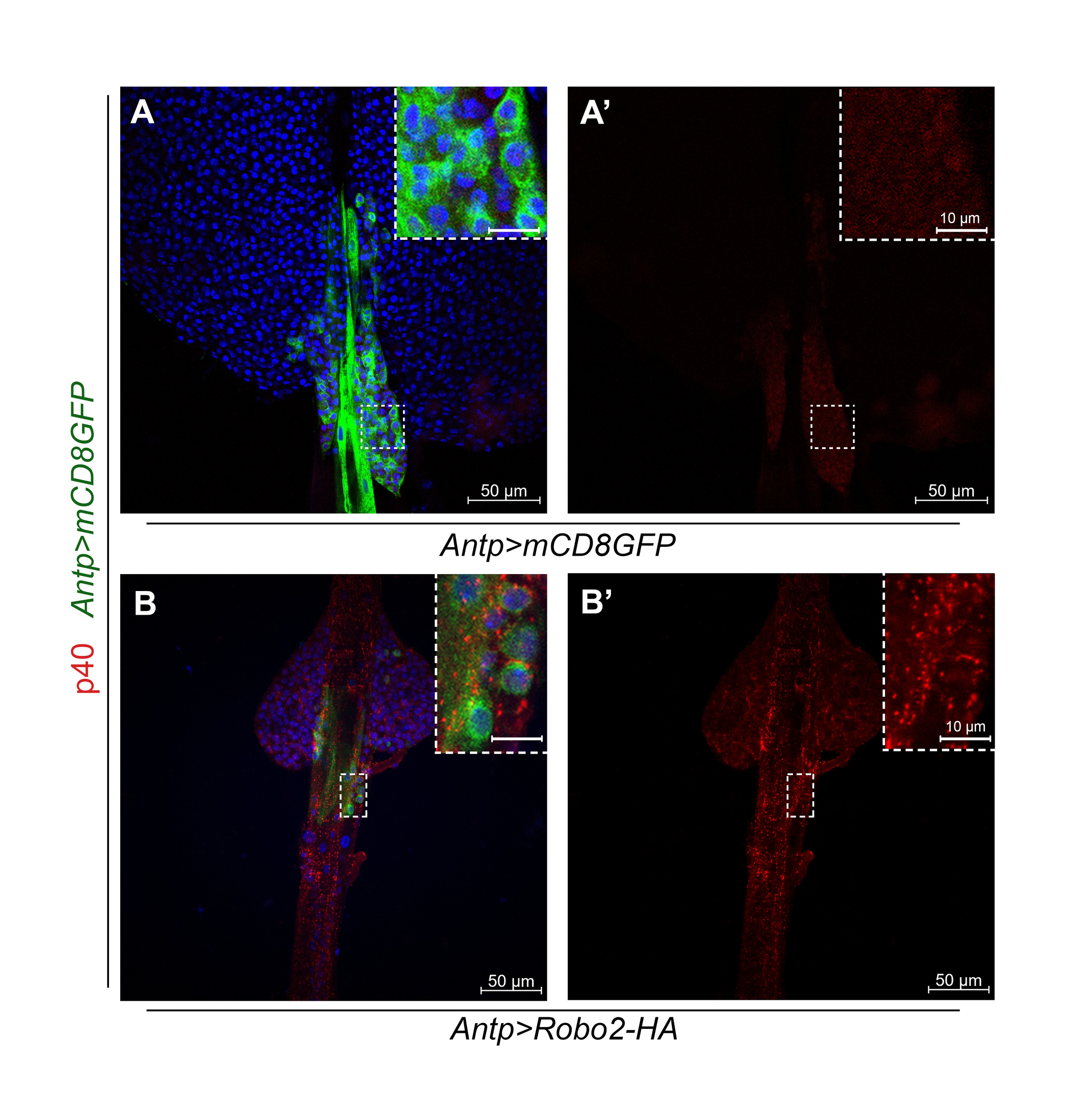

Supplement: S3 Fig — Anterior lobes of lymph glands from uninfected Antp>mCD8GFP (A, A’) and Lh-infected Antp>mCD8GFP, Robo2-HA animals (B, B’). EVs are absent in glands of naïve animals (A, A’) but clearly observed and widely distributed in glands of infected animals. The PSC is no longer tightly clustered. (The sample in panels B, B’ is the same as shown in S2 Fig, panels F, F’). (TIF) [file ppat.1009615.s003.tif]

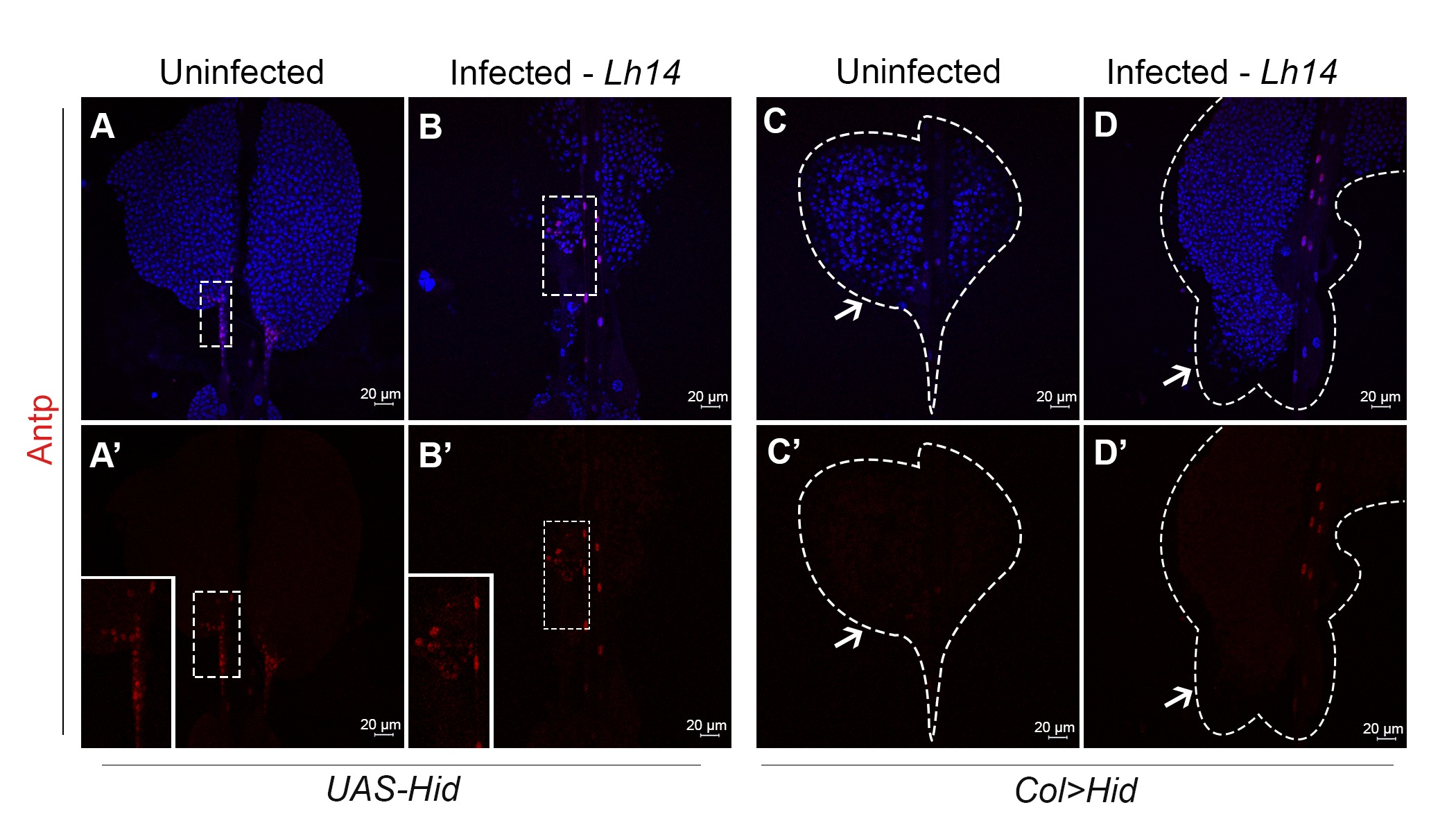

Supplement: S4 Fig — (A, A’) A normal-sized and intact PSC, expresses Antp in UAS-Hid animals. Lobes from naïve animals have normal morphology. (B, B’) An Antp-positive PSC is disassembled in UAS-Hid animals after Lh infection. Lobes are reduced in size. Insets in panels A’ and B’ show Antp-positive PSC cells. (C, D) A PSC-less lymph gland from Col>Hid naïve and Lh-infected hosts. Lobes are Antp-negative. Col>Hid lobes remain intact after Lh-infection (D, D’). The dashed lines in panels (C) and (D) show the areas where biological samples are present. Arrows point to the general locations where the PSCs should have formed. (TIF) [file ppat.1009615.s004.tif]

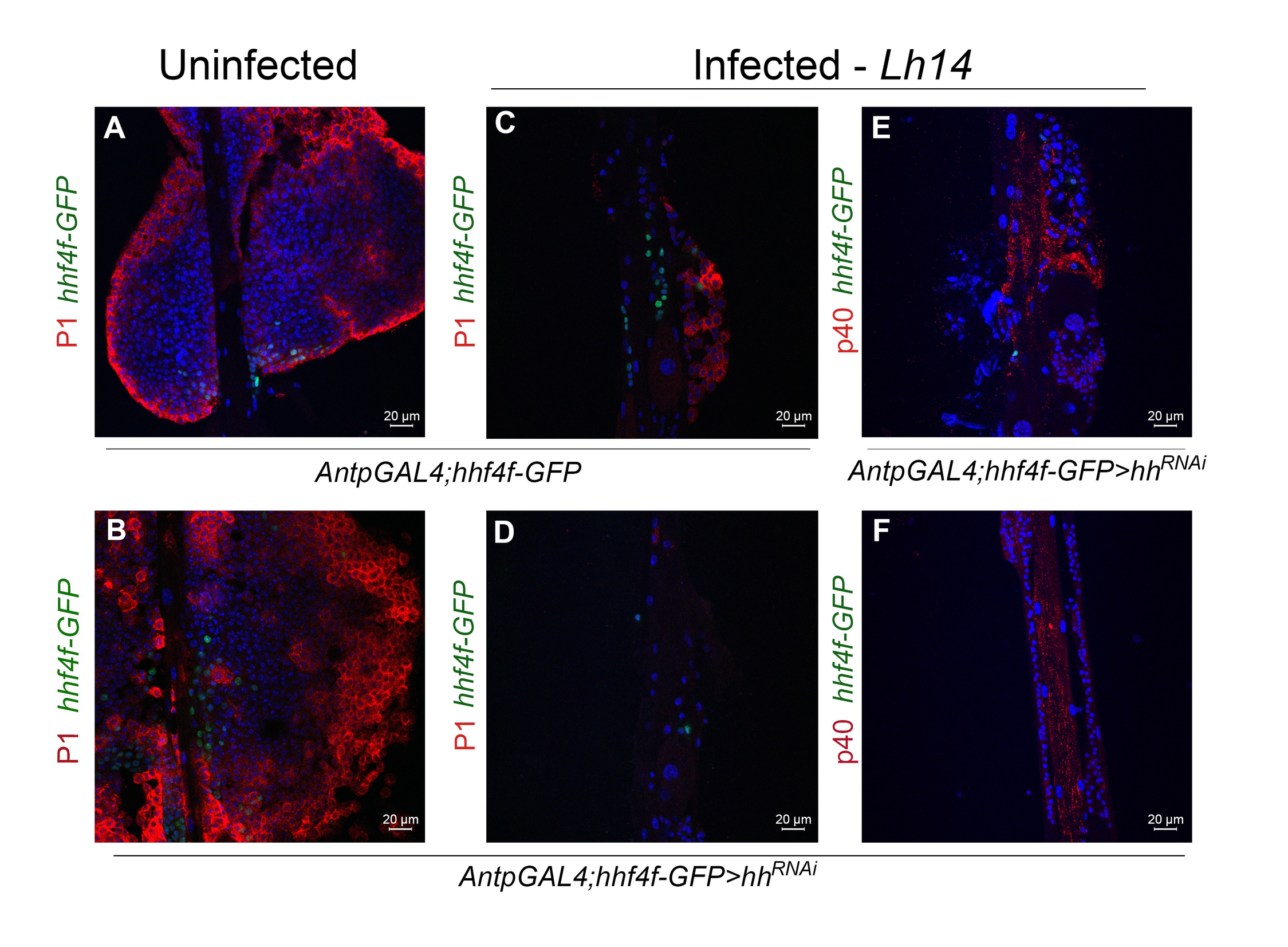

Supplement: S5 Fig — (A-D) Lobes from naïve Antp-GAL4; hhf4f-GFP (A, C) and Antp>hhRNAi; hhf4f-GFP (B, D) hosts. hh KD increased cortical P1-positive cells (B); Lh infection leads to hemocyte loss and disassembled PSCs. P1-positive cells are observed post-infection (D). (E, F) Anterior (E) and posterior (F) lobes from Lh-infected Antp>hhRNAi; hhf4f-GFP hosts show EVs in the few remaining hemocytes. EVs are also evident in the dorsal vessel. (TIF) [file ppat.1009615.s005.tif]

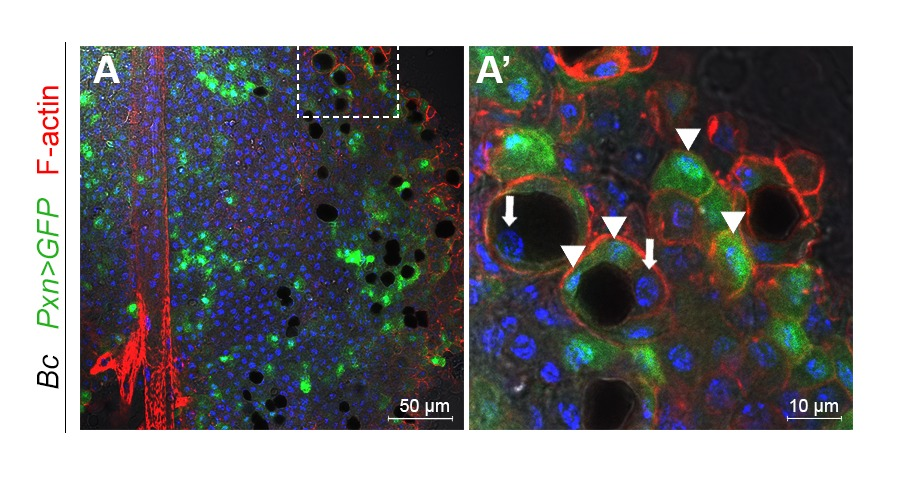

Supplement: S6 Fig — (A, A’) A Bc+/Bc Pxn>GFP gland showing blackened crystal cells within Pxn>GFP-expressing hemocytes. Arrows points to crystal cell nuclei; arrowheads point to Pxn>GFP-positive macrophages. Not all macrophages contain a crystal cell. (TIF) [file ppat.1009615.s006.tif]

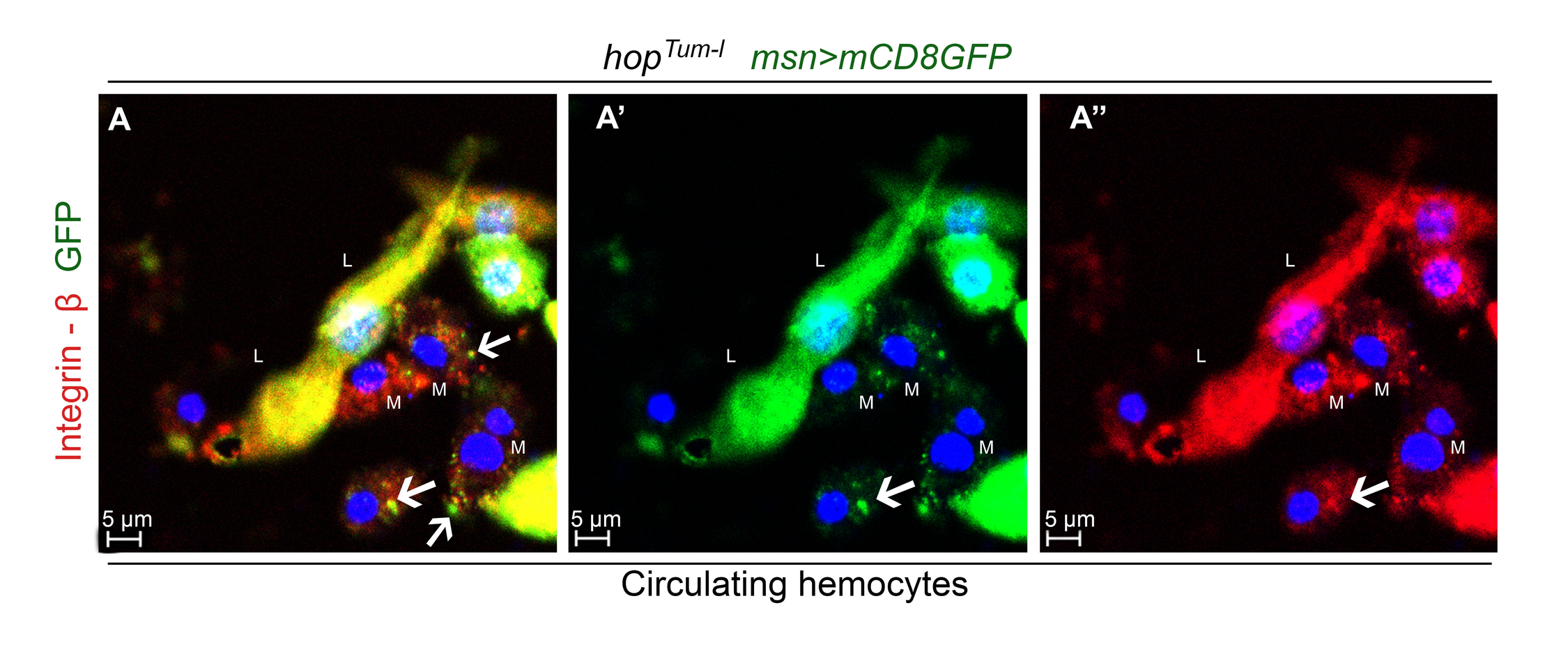

Supplement: S7 Fig — Hemocytes from an Lh-infected hopTum-l host in which lamellocytes (L) express mCD8GFP. Lamellocytes also express high levels of integrin-beta. Double positive lamellocyte fragments in panel A are observed in macrophages (M) indicated by arrows. Signals in A’ and A” are merged in panel A. (TIF) [file ppat.1009615.s007.tif]

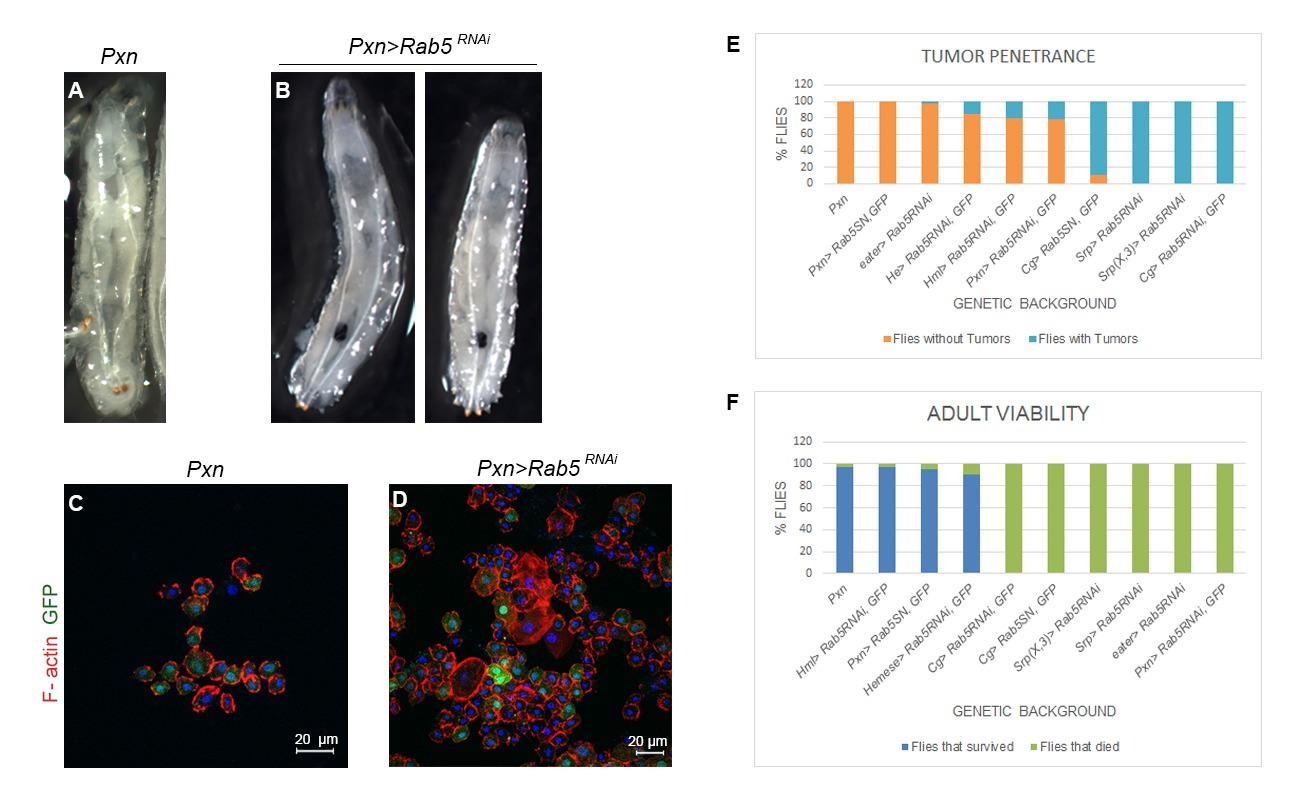

Supplement: S8 Fig — (A, B) Pxn>GFP, Rab5RNAi larvae with melanized tumors. Tumors are absent in the control animal. (C, D) Circulating hemocytes from Rab5 KD animals show an overabundance of Pxn>GFP-positive and GFP-negative (lamellocytes) cells. (E) Tumor penetrance (animals with tumors/animals scored) in Rab5 KD animals varied with different GAL4 drivers. (F) Viability to adulthood was differentially affected. More than 100 animals were scored for each cross in panels (E) and (F). (TIF) [file ppat.1009615.s008.tif]
